# Proportion of neuropathic pain in the back region in chronic low back pain patients -a multicenter investigation

**DOI:** 10.1038/s41598-018-33832-x

**Published:** 2018-11-08

**Authors:** Jun Li, Jing He, Hu Li, Bi-Fa Fan, Bo-Tao Liu, Peng Mao, Yi Jin, Zhu-Qiang Cheng, Ting-Jie Zhang, Zhi-Fang Zhong, Si-Ji Li, Sai-Nan Zhu, Yi Feng

**Affiliations:** 1 Peking University People’s Hospital, Department of Pain Medicine, Beijing, 100044 China; 2 Peking University People’s Hospital, Arthritis Clinical & Research Center, Beijing, 100044 China; 3 China-Japan Friendship Hospital, Department of Pain Medicine, Beijing, 100029 China; 4 Jinling Hospital, Department of Anesthesiology, Pain Medicine Center, Nanjing, 210002 China; 5 Peking University First Hospital, Department of Epidemiology, Beijing, 100034 China

## Abstract

Neuropathy can contribute to low back pain (LBP) in the region of the back. Our study investigated the proportion of neuropathic pain (NP) in low back region in chronic LBP patients from multicenter and clinics in China and identified associated factors. Assessment was made using a questionnaire and the Leeds Assessment of Neuropathic Symptoms and Signs (LANSS, only tested in low back region), as well as Quantitative Sensory Testing (QST, merely applied to the low back region), the Hospital Anxiety and Depression Scale (HADS) and the Oswestry Disability Index (ODI). Our questionnaire collected demographic information, behavioral habits and medical records. 2116 outpatients over 18 years old complaining of LBP lasting more than 3 months were enrolled in this study. The NP proportion in low back region in chronic LBP patients was 2.8%. Multivariable logistic regression analysis showed that histories of lumbar surgery, abdominal or pelvic surgery, and drinking alcohol were independent positive predictors for LBP of predominantly neuropathic origin (LBNPO), while history of low back sprain and frequently carrying weight as independent negative predictor. Using these parameters may help the identification of patients with chronic LBP likely to develop NP leading to improved treatment outcomes.

## Introduction

Low back pain (LBP) is one of the most common diseases occurring with neuropathic pain (NP)^1^. Low back pain of neuropathic origin (LBNPO) often develops into chronic LBP, characterized by a protracted disease course and recurrent attacks. Commonly available therapies are largely ineffective^2–4^. NP are complicated by depression, anxiety and other symptoms^5–10^, imposing a great burden to families and society. Given the prevalence of LBP in many industrial countries, the medical expense caused by NP can account for an important part of the cost of LBP^11–13^. Therefore diagnosis of NP in LBP is important for further effective targeted therapy.

At present, multiple studies have shown that a nociceptive origin is more common in chronic LBP^6,14,15^. Two surveys by the Freynhagen team in Germany found that the prevalence rates of LBP patients with an NP component were 37% and 33.5%, respectively^6,15^. A British study found the proportion of NP in chronic back pain patients selected from the ordinary population was 15.8%^16^. The prevalence of NP among chronic LBP patients in Saudi Arabia were 41% and 54.7%^17,18^, while the prevalence of NP among African patients suffering from common low back pain was 49.5%^19^.

However, the proportion of NP and associated factors in low back region in Chinese LBP patients have not been investigated. If doctors can identify risk factors for NP in LBP, and intervene early, it may decrease the prevalence of NP in LBP, and reduce the medical expenditure. This study aimed to investigate the proportion of chronic LBP patients with NP in low back region (not referring to leg pain) in clinics and looked for associated factors that could have significance in guiding clinical practice.

## Results

### Sample

A total of 2116 pairs of questionnaires were handed out in three research centers. Excluding unqualified patients and those with incomplete data, 1699 pairs of valid questionnaires were obtained, with a total eligible rate of 80.3%. Eligible subjects consisted of 58.2% females and 41.8% males and the average age was 49.4 ± 15.3 years old (ranging from 18 to 91 years old).

### The proportion of LBP patients with NP

Our cohort included a total of 48 cases with LBNPO with Leeds Assessment of Neuropathic Symptoms and Signs (LANSS) scores greater than or equal to 12, giving an NP proportion of 2.8% (95% CI, 2.0–3.6%). Protrusion of the lumbar intervertebral disc and lower back myofascitis were the most common diagnoses for chronic LBP (Table 1). Two or more different diagnoses may appear simultaneously in the same patients. For example, there were 27 patients suffering from protrusion of the lumbar intervertebral disc and lumbar spinal stenosis, 17 patients bearing protrusion of lumbar intervertebral disc and lower back myofascitis, and 4 patients tolerating the above three diseases at the same time. Other diagnoses involved in this study included lumbar vertebral compression fracture, sacroiliitis, the third lumbar vertebral transverse process syndrome etc.

**Table 1:** Proportion of NP* in common low back diseases.

Diagnosis	Total case	NP case	NP proportion	95% CI
Low back myofascitis	764	7	0.9%	0.2–1.6%
Protrusion of lumbar intervertebral disc	572	16	2.8%	1.4–4.2%
Lumbar spinal stenosis	79	5	6.3%	0.8–11.8%
Lumbar facet joint syndrome	86	1	1.2%	0.1–3.5%
Failed back surgery syndrome	16	3	18.8%	5.0–41.7%
Others	234	20	8.5%	4.9–12.2%

^*^NP: neuropathic pain.

### ***A***ssociated factors analysis of NP in LBP

Because the number of LBNPO patients (48 cases) was far less than the non-LBNPO patients, we matched 141 non-LBNPO cases (accounting for 8.5% of 1651 cases) according to the age and gender of LBNPO patients. Since no matched data was found for a 91 years old LBNPO patient, this case was excluded from the analysis. Therefore, statistical analysis was carried out with 188 patients (47 LBNPO patients versus 141 non-LBNPO patients).

#### Demographic data

None of the collected demographic data showed statistically significant differences between LBNPO and non-LBNPO patients (the details can be found in Supplementary Table S1). Especially, no statistically significant differences were found between LBNPO and non-LBNPO patients in education degree, occupation, marital status, body mass index (BMI) or household monthly income per person. The classification of household monthly income per person was based on the individual income tax cut-off point in 2009 in China. In addition, having immediate family members suffering from similar LBP, or recent significant life changes were not relevant to the identification of LBP patients with an NP component.

#### Behavioral habits, accompanying diseases and surgery history

In terms of behavioral habits, drinking, smoking, frequently carrying weight were related to LBNPO (Table 2). The total sitting time per day did not differ significantly between LBNPO and non-LBNPO patients, nor was there any significant difference in exercise time (median score: 3.0 hours vs. 4.0 hours, interquartile range (IQR): 0.0–8.0 vs 0.0–10.0, P = 0.825).

**Table 2:** Effect of behavioral habits and medical conditions on NP.

	Non-LBNPO (n = 141)		LBNPO* (n = 47)		P value
	n	%	n	%	
Smoking	26	18.4%	16	34.0%	0.042
Drinking	55	39.0%	27	57.4%	0.041
Frequently bear a heavy weight over 5 kg	70	49.6%	14	29.8%	0.027
Average sitting duration per day(<4 h)	80	56.7%	23	48.9%	0.399
Previously low back sprain	33	23.4%	2	4.3%	0.004
Previously bore a heavy weight	45	31.9%	8	17.0%	0.061
Lumbar surgery	5	3.5%	7	14.9%	0.012
Abdominal/pelvic surgery	8	5.7%	9	19.1%	0.015
Diabetes mellitus	19	13.5%	7	14.9%	1.000
Hypertension	19	13.5%	11	23.4%	0.166

^*^LBNPO: low back pain of neuropathic origin.

Besides, our data indicated that a previous histories of lumbar, abdominal or pelvic surgery were associated with LBNPO (Table 3). And patients with low back sprain had a significantly lower probability of developing LBNPO than LBP of predominantly nociceptive type. In patients with hypertension or diabetes mellitus, the probability of developing LBNPO was not significantly different from that of non-LBNPO. There was no obvious correlation between a heavy load bearing history and LBNPO.

**Table 3:** Associated factors for LBNPO.

	ODDS RATIO^a^ (95% CI)	P value	ODDS RATIO^b^ (95% CI)	P value
Lumbar surgery	4.76 (1.43, 15.81)	0.011	3.87 (1.12, 13.33)	0.032
Abdominal/pelvic surgery	3.94 (1.42–10.90)	0.008	3.39 (1.16–9.91)	0.026
Drinking	2.11 (1.08,4.12)	0.029	2.15 (1.04, 4.44)	0.039
Frequently bear a heavy weight over 5 kg	0.43 (0.21–0.87)	0.019	0.46 (0.22–0.99)	0.047
Previously low back sprain	0.14 (0.03–0.63)	0.01	0.15 (0.03, 0.71)	0.016
Smoking	2.28 (1.09–4.78)	0.028	—	

^a^Univariate binary logistic analysis result.

^b^Multivariate binary logistic analysis result.

#### Associated factor analysis

Univariate and multivariate binary logistic regression analyses were carried out using the above mentioned significant factors between the two patient populations. Our results showed that previous histories of lumbar surgery, abdominal or pelvic surgery, drinking alcohol could be used as independent positive predictors for LBNPO, while a history of low back sprain and frequently carrying weight were the independent negative predictors (Table 3).

#### Pain, psychology, vital functions and disease burden

Pain: There was no significant difference between LBNPO (median score 15.0 months, IQR 10.0 to 36.0) and non-LBNPO patients (median score 11.0 months, IQR 4.0 to 36.0; P = 0.129) in the pain duration. The average pain intensity score was significantly higher (mean score 5.72, SD 1.81) in LBNPO patients than in non-LBNPO patients (mean score 4.58, SD 1.93; P < 0.001).

Psychological state: There were a total of 112 qualified patients among those completing Hospital Anxiety and Depression Scale (HADS, including 23 cases with LANSS ≥12, accounting for 47.9% of the 48 LBNPO cases; and 88 cases with LANSS <12, accounting for 5.3% of the 1651 cases). The percentage of anxiety and suspected anxiety were significantly higher in LBNPO patients than those non-LBNPO patients (65.2% vs 36.4%, P = 0.010, see Supplementary Table S3). There was also a significant difference between LBNPO and non-LBNPO patients in the percentage of depression and suspected depression (52.1% vs 27.3%, P = 0.046).

Physical disability: There were a total of 123 qualified patients among those filling out Oswestry Disability Index (ODI, including 26 cases with LANSS ≥12, accounting for 54.2% of the 48 LBNPO cases; and 97 cases with LANSS <12, accounting for 5.9% of the 1651 cases). The disability index was significantly higher in LBNPO patients than that of non-LBNPO patients (42.1% ± 19.7% vs 25.8% ± 15.5%, p < 0.001).

Disease burden: When we compared the disease burden between LBNPO and non-LBNPO patients, LBNPO patients visited the doctor significantly more frequent than non-LBNPO patients. The time off work attributed to LBP was significantly longer in LBNPO patients than non-LBNPO patients and 59.6% of LBNPO patients asked for leave more than 30 days. In all patients seeking medical treatment, the medical expenditure for LBNPO patients was significantly higher than that of non-LBNPO patients. Approximately 70% of non-LBNPO patients spent less than 4000 RMB, while approximately 70% of LBNPO patients spent more than 4000 RMB (Tables 4 and 5).

**Table 4:** Frequency of medical visits and medical leave for LBNPO and non-LBNPO patients.

	Non-LBNPO (n = 141)		LBNPO (n = 47)		P value
	n	%	n	%	
Medical visit					P < 0.001
Never	48	34.0%	2	4.3%	
1–5	57	40.4%	24	51.1%	
6–7	19	13.5%	6	12.8%	
>10	17	12.1%	15	31.9%	
Medical leave					P < 0.001
Never	87	61.7%	7	14.9%	
1~3 days	12	8.5%	6	12.8%	
4~7 days	6	4.3%	0	0.00%	
8~14 days	7	5.0%	2	4.3%	
15~30 days	6	4.3%	4	8.5%	
≥30 days	23	16.3%	28	59.6%

**Table 5:** Medical Expenditure for LBNPO and non-LBNPO patients.

	non-LBNPO (n = 93)		LBNPO (n = 45)		P value
	n	%	n	%	
Medical Expenditure (RMB)					P < 0.001
<1000	30	32.3%	4	8.9%	
1000–4000	31	33.3%	10	22.2%	
4000–7000	11	11.8%	2	4.4%	
7000–10000	5	5.4%	6	13.3%	
≥10000	16	17.2%	23	51.1%	

Quantitative Sensory Testing (QST): There was no obvious difference between LBNPO patients and non-LBNPO patients with regards to the values of QST (Tables 6 and 7).

**Table 6:** QST^a^ results for LBNPO and non-LBNPO patients (CDT^b^, WDT^c^, CPT^d^, HPT^e^).

	non-LBNPO (n = 91) Mean ± SD	LBNPO (n = 23) Mean ± SD	P value
Average of CDT	30.2 ± 1.2	28.5 ± 5.8	0.290
Average of WDT	34.5 ± 0.97	35.5 ± 2.2	0.058
Average of CPT	18.1 ± 10.9	21.1 ± 10.8	0.070
Average of HPT	41.2 ± 3.8	40.0 ± 4.4	0.125

^a^QST: Quantitative sensory testing.

^b^CDT: Cold detection threshold.

^c^WDT: Warm detection threshold.

^d^CPT: Cold pain threshold.

^e^HPT: Heat pain threshold.

**Table 7:** Average VDT* results for LBNPO and non-LBNPO patients.

	non-LBNPO (n = 91)		LBNPO (n = 23)		P value
	Median	Quantile	Median	Quantile	
Average VDT	12.2	6.1, 22.8	8.6	5.4, 16.3	0.274

^*^VDT: Vibration detection threshold.

## Discussion

In this study, we surveyed demographics, behavioral habits, and medical histories of chronic LBP patients in three large general hospitals in China and screened out LBNPO patients using the LANSS questionnaire. We calculated the proportion of NP in chronic LBP and common LBP diseases. In addition, we identified associated factors for NP in chronic LBP and compared the emotional state (HADS), physical disability (ODI) and nerve fiber function (QST) between LBNPO and non-LBNPO patients.

The proportion of chronic LBP patients with NP was calculated to be 2.8%, far less than the results from other countries (e.g. Germany, UK, Saudi Arabia, ranging from 15.8–54.7%)^15,16,18^. We speculated that this lower percentage might be due to regional or ethnic differences, differences in the location of pain, methodology, inclusion/exclusion criteria, cultural and genetic background, or the use of different screening tools^17,20,21^. In the study applying LANSS, Kaki *et al*. found a prevalence of 54.7% in Saudi Arabia^18^ and Hassan *et al*. reported prevalence of 41% in a pilot study in the same country^17^. Torrance *et al*. in the UK discovered a prevalence of 15.8% by using s-LANSS^16^. To establish a Chinese version of LANSS scale, forward translation, backward translation, pilot testing and pain specialists’ evaluations were conducted^22^. The reliability (Cronbachi’s alpha coefficient and Guttman split-half coefficient >0.7) and validity (sensitivity: 80.0%, specificity: 97.1%, positive and negative predictive values: 96.6% and 82.9%, receiver operating characteristic [ROC] curve and the area under the ROC curve: 0.963 ± 0.015) were reasonable. The sensitivity of the Chinese version was slightly lower than the English version (sensitivity and specificity: 83% and 87%)^23^. And the mean value of the area under the ROC curve of the Chinese version was larger than 0.9, indicating its high diagnostic value.

A key finding of our study was that a previous history of lumbar surgery was the most significant associated factor for LBNPO. This was consistent with those reports that damage to the local nerve root or postoperative scar may result in neuropathic pain in the low back^24^. Another possible explanation is that NP is related to granulomatous lesions of a degenerative intervertebral disc^14,25,26^. NP may also be the cause of lumbar surgery and therefore it is difficult to identify the direction of causation. The statistical analysis also showed that a previous history of abdominal or pelvic surgery was an associated factor for LBNPO. We supposed that abdominal or pelvic surgery may cause peripheral nerve injury, lead to central sensitization and therefore cause the central NP in later instances of LBP.

Alcohol was found to be an associated factor for LBNPO. There were numerous evidences that alcohol was related to peripheral neuropathy due to thiamine deficiency^27–31^. Recent investigations in alcohol-induced neuropathologies have revealed that the expression of a variety of inflammatory molecules is increased in the central nervous system in adult alcoholics^32–34^. We speculated that alcohol may be the risk factor for LBNPO. A previous history of low back sprain was negatively correlated to LBNPO. Frequently carrying a heavy weight over 5 kg was also an associated factor to non-LBNPO. The heavy physical work may cause damages to the muscles, fascia and ligaments.

In this study, we found that LBNPO was associated with anxiety and depression. Anxiety and depression were risk factors for LBP^35^ and also related to NP^6,7^. They may be the risk factors for LBNPO. The severe pain might affect the psychological state of patients. Meanwhile, we found that the medical expenditure of LBNPO patients may cause a heavier social burden, which was consistent with the results reported by Schmidt *et al*. and Freynhagen *et al*.^14,36^. We also noticed that the degree of disability was significantly higher in LBNPO patients than non-LBNPO patients, which may further aggravate the disease burden.

QST is an auxiliary way to diagnose NP with 13 items; approximately 92% of patients with NP have at least one abnormal test result^37^. In this study, we found no statistically significant differences in the results of cold detection threshold (CDT), warm detection threshold (WDT), cold pain threshold (CPT), hear pain threshold (HPT) and vibration detection threshold (VDT) between LBNPO and non-LBNPO patients, suggesting that the QST of chronic LBP of predominantly nociceptive pain also had abnormalities or cutaneous sensation on the waist was insensitivity. The difference in lumbar QST between LBP patients and normal controls needs to be further investigated Previous studies showed that almost all of QST methods (except for the wind-up ratio and dynamic mechanic allodynia) were able to discriminate between the painful diabetic neuropathy patients and healthy controls^38^. Furthermore, there were significant differences in the QST between NP and non-NP in the diabetic neuropathy screened by LANSS scale^39^. In our study, no difference in QST between LBNPO and non-LBNPO was found. We therefore speculated that the QST contribute little to the diagnosis of LBNPO. Furthermore, according to the results of QST, the Chinese version of LANSS scale might not be sensitive enough to assess NP in the low back region among chronic LBP patients despite its reasonable reliability and validity.

Several limitations existed in our study. Firstly, we diagnosed the neuropathic pain by applying LANSS and testing the most painful area in low back. But we did not collect any information about leg pain, including both characteristics and topography. However, neuropathic mechanisms may play a more important role in leg pain^6,14,15^. Secondly, this study was an observational cross-sectional study. This study design could not yield the highest level of evidence as randomized trials and therefore we were unable to draw causal inferences in our association analysis. Thirdly, the small sample size of the association study might have influences on the result. Finally, all three research centers controlled the selection bias by strictly applying the patient inclusion and exclusion criteria. But since the referral procedure was imperfect in China, patients could visit a large general hospital directly without visiting a community hospital first. Consequently the intractable cases could be diluted, which may further lead to the lower NP proportion.

In conclusion, the proportion of chronic LBP outpatients with NP in Chinese hospitals was 2.8%. Previous histories of lumbar surgery, abdominal or pelvic surgery and drinking alcohol were independent positive predictors for chronic LBNPO, while frequently bearing a weight over 5 kg and previously low back sprain were independent negative predictors for chronic LBNPO. Using these parameters may help with the identification of chronic LBP patients who are likely to develop NP and may lead to improved treatment outcomes.

## Methods

### Subjects

This multicenter cross-sectional study was reviewed and approved by the Ethics Committee of Peking University People’s Hospital. In this study, 2116 outpatients with chronic LBP were recruited from three research centers in 2 Chinese cities (Peking University People’s Hospital and China-Japan Friendship Hospital in Beijing, Jinling Hospital in Nanjing) during the period between January, 2012 and November, 2014. The patient inclusion criteria were: age ≥ 18 years, fulfilling the chronic LBP diagnosis, with stable mental health, and the ability to give written informed consent. Patients with schizophrenia or with central nerve system disorders (e.g. myelitis, cerebral hemorrhage, cerebral infarction and multiple sclerosis) were excluded from this study. All methods were performed in accordance with the relevant guidelines and regulations. All patients gave written informed consent for participation.

Sample size(n) was estimated under the assumption of 6% NP prevalence (P) in chronic LBP patients^36^, 2% permissible error (δ) between sample and population, 5% Type I error rate, and U_0.05_ at 1.96 according to the formula n = (U_α_/δ)/P(1 − P)^40^. An appropriate sample size of 1738 patients was required for this study. Considering that 20% of participants might present with incomplete data, the target sample size was set at 2086 patients.

All qualified patients were invited to complete two questionnaires. One was a questionnaire developed by our group, collecting demographic information, behavioral habits and medical records. The other was a Chinese version of LANSS^22^, which was used for assessing neuropathic pain. LANSS scale consists of seven questions, among which five are about symptoms (i.e. dysesthesia, autonomic dysfunction, evoked pain, paroxysmal pain and thermal pain respectively). Some involve couple of descriptors. For example, the strange/unpleasant sensations consist of words like pricking, tingling, pins and needles. Trained clinicians read the questionnaire, asked patients whether the description matched their low back pain characteristics in the preceding week, and then filled the questionnaire. Two examination items are included: the response to lightly stroking cotton wool across the non-painful and the painful area and the raised or lowered pin-prick threshold. The examinations were performed by investigators and were applied to the most painful area in the low back. A LANSS score of 12 or more has been found to have a positive predictive value for neuropathic pain. Patients with LANSS score ≥ 12 points, and randomly chosen subjects with LANSS score < 12 points filled out HADS and ODI and received QST after fundamental physical examination. Considering diagnostic therapy arranged for the patients, QST was conducted in the most painful area of the low back right after completing the LANSS scale so as to acquire the data of QST before therapy. Random selection was performed as follows: subjects with LANSS score < 12 points were divided into groups of eight in chronological order of enrolling. Then block randomization was applied for each group. A total of five variables were included in the QST in this study: CDT, WDT, CPT, HPT, and VDT. The QST was performed with a TSA-II Quantitative NeuroSensory Analyzer (Medoc, Israel), to measure the thresholds of thermal sensation, thermal pain sensation and vibration detection at the most painful region of the low back with Limits method. The baseline temperature was 32 °C. Temperature change was 1 °C per second and the cut-off limits 0 °C and 50 °C. The contact area of the thermode was 9 cm^2^. Thresholds of the thermal sensation were obtained immediately when the subject felt cold or warm and pressed a button while thresholds of the thermal pain sensation were obtained immediately when the subject felt pain and pressed a button. The mean thresholds for cold, warm, cold pain and heat pain were determined in three tests and set as the final threshold. Meanwhile, the mean threshold of 5 tests was used as the final threshold for vibration.

### LBP diagnosis

Low back pain is usually defined as pain, muscle tension, or stiffness localized below the costal margin and above the inferior gluteal folds, with or without leg pain^35^. Chronic LBP refers to LBP lasting more than 3 months. In order to maximize the accuracy of diagnosis, diagnosis was independently given by two attending physicians according to the internationally recognized diagnostic criteria.

### Statistical analysis

Data was analyzed with SPSS for Windows 19.0 (SPSS, Inc., Chicago, IL). Measurement data were described as mean ± standard deviation or median value (quartiles). Qualitative and ranked data were expressed by case and percentage. Student’s t test or Wilcoxon rank sum test was used for measurement data, while χ^2^ test or Fisher’s exact test was used for qualitative data. The Wilcoxon rank sum test was used for ranked data. Univariate and multivariate binary logistic regressions were applied to analyzing the associated factors for NP involvement in LBP patients. To calculate the OR value and its 95% confidence interval, multifactor screening with selected backward method was used for analysis. All tests were two-tailed, with p < 0.05 set as the indicator of statistically significance.

## Electronic supplementary material

supplementary information

## Data Availability

All data generated or analysed during this study are included in this published article (and its Supplementary Information files).
