# Supplementary material for: Proportion of neuropathic pain in the back region in chronic low back pain patients -a multicenter investigation
Source: Sci Rep. 2018 Nov 8;8:16537. doi: 10.1038/s41598-018-33832-x (PMC6224392; doi:10.1038/s41598-018-33832-x)
Supplement: Supplementary file 1 — supplementary information [file 41598_2018_33832_MOESM1_ESM.docx]

**Proportion of neuropathic pain in the back region in chronic low back pain patients -a multicenter investigation**

LI Jun ^1+^, HE Jing ^1+^, LI Hu ^2^, FAN Bi-Fa ^3^, LIU Bo-Tao ^3^, MAO Peng ^3^, JIN Yi ^4^, CHENG Zhu-Qiang ^4^, ZHANG Ting-Jie ^1^, ZHONG Zhi-Fang ^1^, LI Si-Ji ^1^, ZHU Sai-Nan ^5^, FENG Yi ^1*^

^1^ Peking University People’s Hospital, Department of Pain Medicine, Beijing, 100044, China

^2^ Peking University People’s Hospital, Arthritis Clinical & Research Center, Beijing, 100044, China

^3^ China-Japan Friendship Hospital, Department of Pain Medicine, Beijing, 100029, China

^4^ Jinling Hospital, Department of Anesthesiology, Pain Medicine Center, Nanjing, 210002 China

^5^ Peking University First Hospital, Department of Epidemiology, Beijing, 100034, China

*Corresponding author. [Tel.:010-8832](mailto:Tel.:010-8832)5590; E-mail address: [yifeng65@sina.com](mailto:yifeng65@sina.com) (Y. Feng).

^+^these authors were co-first authors, they contributed equally to this work.

Supplementary Table S1. Demographic data of patient cohort, and association with NP

| Demographic data | Non-LBPNO (n = 141) | | LBPNO (n = 47) | | P value |
| --- | --- | --- | --- | --- | --- |
|  | n | % | n | % |  |
| Education degree |  |  |  |  | 0.122 |
| Illiteracy, semiliteracy | 2 | 1.4% | 1 | 2.1% |  |
| Senior high school or below | 66 | 46.8% | 29 | 61.7% |  |
| Junior college or above | 73 | 51.8% | 17 | 36.2% |  |
| Occupation |  |  |  |  | 0.609 |
| Worker | 35 | 24.8% | 15 | 31.9% |  |
| Farmer | 6 | 4.3% | 3 | 6.4% |  |
| soldier | 2 | 1.4% | 1 | 2.1% |  |
| Driver | 6 | 4.3% | 1 | 2.1% |  |
| Doctor | 14 | 9.9% | 9 | 19.1% |  |
| Teacher | 25 | 17.7% | 6 | 12.8% |  |
| staff | 8 | 5.7% | 1 | 2.1% |  |
| Retiree | 7 | 5.0% | 2 | 4.3% |  |
| Others | 38 | 27.0% | 9 | 19.1% |  |
| Marital status |  |  |  |  | 0.970 |
| spinsterhood | 10 | 7.1% | 4 | 8.5% |  |
| married | 120 | 85.1% | 40 | 85.1% |  |
| divorced | 3 | 2.1% | 1 | 2.1% |  |
| widowed | 8 | 5.7% | 2 | 4.3% |  |
| Family monthly income per person (RMB) |  |  |  |  | 0.512 |
| <2000 | 32 | 22.7% | 6 | 12.8% |  |
| 2000-5000 | 78 | 55.3% | 27 | 57.4% |  |
| 5000-20000 | 28 | 19.9% | 12 | 25.5% |  |
| >=20000 | 3 | 2.1% | 2 | 4.3% |  |
| Immediate family members suffering from LBP | 63 | 44.7% | 18 | 38.3% | 0.499 |
| Recently occurred any significant misfortune | 7 | 5.0% | 4 | 8.5% | 0.472 |
| BMI |  |  |  |  | 0.541 |
| Underweight (<18.5) | 4 | 2.8% | 2 | 4.3% |  |
| Normal weight (18.5 – 24) | 68 | 48.2% | 26 | 55.3% |  |
| Overweight and obese (≥24) | 69 | 48.9% | 19 | 40.4% |  |

Supplementary Table S2. BMI of patient cohort, associated with NP

|  | non-LBNPO （n = 141）  Mean ± SD | LBNPO （n = 47）  Mean ± SD | P value |
| --- | --- | --- | --- |
| BMI | 24.0 ± 3.4 | 23.0 ± 3.2 | 0.101 |

Supplementary Table S3. HADS between LBNPO and non-LBNPO

|  | Non-LBNPO(n=88) | | LBNPO(n=23) | | P value |
| --- | --- | --- | --- | --- | --- |
|  | n | % | n | % |  |
| Anxiety score |  |  |  |  | 0.010 |
| <=7 | 56 | 63.6% | 8 | 34.8% |  |
| 8-10 | 22 | 25.0% | 7 | 30.4% |  |
| >10 | 10 | 11.4% | 8 | 34.8% |  |
| Depression score |  |  |  |  | 0.046 |
| <=7 | 64 | 72.7% | 11 | 47.8% |  |
| 8-10 | 17 | 19.3% | 7 | 30.4% |  |
| >10 | 7 | 8.0% | 5 | 21.7% |  |
